# Comorbidity of depression and anxiety leads to a poor prognosis following angina pectoris patients: a prospective study

**DOI:** 10.1186/s12888-021-03202-5

**Published:** 2021-04-20

**Authors:** Bingqing Bai, Han Yin, Lan Guo, Huan Ma, Haochen Wang, Fengyao Liu, Yanting Liang, Anbang Liu, Qingshan Geng

**Affiliations:** 1 Guangdong Cardiovascular Institute, Guangdong Provincial People’s Hospital, Guangdong Academy of Medical Sciences, No.106 Zhongshan Er Road, Yuexiu District, Guangzhou, 510080 People’s Republic of China; 2 School of Medicine, South China University of Technology, Guangzhou, People’s Republic of China; 3 Department of Cardiac Rehabilitation, Guangdong Cardiovascular Institute, Guangdong Provincial People’s Hospital, Guangdong Academy of Medical Sciences, No.106 Zhongshan Er Road, Yuexiu District, Guangzhou, 510080 People’s Republic of China; 4 Guangdong Provincial People’s Hospital, Guangdong Academy of Medical Sciences, Guangdong Cardiovascular Institute, No.106 Zhongshan Er Road, Yuexiu District, Guangzhou, 510080 People’s Republic of China

**Keywords:** Depression, Anxiety, Comorbidity, Angina pectoris, Prognosis, Cardiovascular disease

## Abstract

**Background:**

Depression and anxiety are two common mood problems among patients with cardiovascular disease (CVD) and are associated with poor cardiac prognoses. The comorbidity of depression and anxiety is considered to be a more severe psychological status than non-comorbid mood disorders. However, little is known about the relationship between depression or anxiety and noncardiac readmission. We conducted a prospective study on the prognostic impact of depression, anxiety, and the comorbidity of the two among angina pectoris (AP) patients.

**Method:**

In this prospective study, 443 patients with AP were included in the analysis. Follow-up assessments were performed 1 year, and 2 years after patient discharges. Clinical outcomes of interest included noncardiac readmission, major adverse cardiovascular events (MACEs), and composite events. Depression and anxiety symptom scores derived from the patient health questionnaire-9 (PHQ-9) and generalised anxiety disorder-7 (GAD-7) questionnaire were used to assess mood symptoms at baseline. Participants with symptom scores of ≥10 on both the depression and anxiety questionnaires formed the clinical comorbidity subgroup. We used multivariable Cox proportional hazards models to evaluate the impact of individual mood symptom and comorbidity on clinical outcomes.

**Results:**

Among all the AP patients, 172 (38. 9%) were determined to have depression symptoms, 127 (28.7%) patients had anxiety symptoms and 71 (16.0%) patients suffered from their comorbidity. After controlling covariates, we found that patients who endured clinical depression (hazard ratio [HR] = 2.38, 95% confidence interval [CI] 1.06–5.33, *p* = 0.035) and anxiety ([HR] 2.85, 95% [CI] 1.10–7.45, *p* = 0.032) had a high risk of noncardiac readmission. Compared to participants with no mood symptoms, those with clinical comorbidity of depression and anxiety presented a greater risk of noncardiac readmission ([HR] 2.91, 95% [CI] 1.03–8.18, *p* = 0.043) MACEs ([HR] 2.38, 95% [CI] 1.11–5.10, *p* = 0.025) and composite event ([HR] 2.52, 95% [CI] 1.35–4.69, *p* = 0.004).

**Conclusion:**

Depression and anxiety were found to have predictive value for noncardiac readmission among patients with AP. Furthermore, prognoses were found to be worse for patients with comorbidity of depression and anxiety than those with single mood symptom. Additional attention needs to be focused on the initial identification and long-term monitoring of mood symptom comorbidity.

**Supplementary Information:**

The online version contains supplementary material available at 10.1186/s12888-021-03202-5.

## Background

Depression and anxiety have been linked to increased risk of coronary heart disease (CHD) and poor prognoses among patients with established cardiovascular disease [1–7]. Although the traditional risk factors for the development and prognosis of CHD are well known, psychosocial factors, especially depression and anxiety, are increasingly recognised [8]. Many studies have shown that depression is associated with a twofold to threefold increase in mortality or the risk of nonfatal cardiac events among patients with diagnosed CHD [9–11]. Anxiety is also an important predictor of recurring CV events among patients diagnosed with myocardial infarction or unstable AP [12–14]. With recurring CV events, the incidence of readmission also rises, which increases the cost of medical care and morbidity rates and affects the quality of life of patients [15]. Although the authors of many related studies paid attention to CVD, such as myocardial infarction or heart failure, and mainly focused on mortality or cardiac-related prognosis [16], there is a paucity of depression and anxiety research concentrated on the population with AP and noncardiac recurrent events.

Depression and anxiety often cooccur. Some studies have shown that there is a high degree of comorbidity between depression and anxiety [17–19], and this comorbidity may increase the risk of all-cause mortality. Patients with this comorbidity experienced greater reductions in functional status than patients with only one of these disorders. It has also been suggested that patients with both depression and anxiety are at an increased risk of CVD [20]. However, few researchers have studied the population with both depression and anxiety and the association between this comorbidity with noncardiac readmission and MACEs.

We conducted a prospective cohort study to measure the prognostic impact of depression and anxiety on 443 AP patients. Our main aim was to examine the effects of depression, anxiety and their comorbidity on noncardiac readmission in AP patients. We also analysed whether minor (scores of ≥5) and major (scores of ≥10) depression or anxiety symptoms, as assessed on PHQ-9 and GAD-7 scales, can be used to predict the occurrence of MACEs and composite events during a 29-month follow-up period after hospitalisation for AP.

## Materials and methods

### Study design and sample

In this prospective study, we investigated the association between depression, anxiety, comorbidity and the occurrence of hospital readmission and recurrent CV events during a 29-month follow-up period among AP patients. Patients were admitted to Guangdong Provincial People’s Hospital from October 2017 to January 2018 for a cross-sectional study and were followed up with every year to observe their prognosis after discharge. Professional cardiologists in the hospital persistently incorporated 705 patients with suspected CHD into the study. All patients underwent examinations and therapies considered to be the most appropriate by the attending cardiologist. A total of 705 copies of the questionnaires were issued, and all the completed copies were retrieved. A well-trained psycho-cardiologist explained the psychiatric scales to each patient 1 day before the coronary angiography and assisted those with impaired vision or poor reading ability to understand the contents of the scale. The study was approved by the Medical Ethics Committee of Guangdong Provincial People’s Hospital. Written informed consent was obtained from all participants. Details about our recruitment strategy and the excluded patients were described in our previous article [21]. A total of 443 individuals with AP (according to the Braunwald standard) and diagnosed CHD were included for analysis.

### Follow-up

Follow-up assessments were performed by telephone at 1 year and over 2 years after discharges. The average follow-up time was 26.33 ± 0.93 months. Endpoint events included all-cause mortality, cardiac and noncardiac readmission, nonfatal stroke, revascularisation and the recurrence of nonfatal myocardial infarction. Noncardiac readmission refers to noncardiac diseases that require the patient to be readmitted for further treatment. MACEs consisted of death, nonfatal stroke, cardiac readmission, revascularisation and the recurrence of nonfatal myocardial infarction. Moreover, the composite event covered all above events. Due to the insufficient samples of death, nonfatal stroke, revascularisation and recurrence of nonfatal myocardial infarction, we did not analyse these clinical events individually.

### Depression and anxiety measures

Patient health questionnaire-9 (PHQ-9) and generalised anxiety disorder-7 (GAD-7) scales were used to assess depression and anxiety at baseline, respectively. We thoroughly elaborated on the utility of these scales in our previous article [21]. In our main analysis, a score of 5 and 10 was used as the mood symptom and clinical mood disorder thresholds. PHQ-9 has been demonstrated to be a dependable measure to evaluate depression severity with mild, moderate, moderately severe to severe depression corresponding to a score of 5, 10 and 15, respectively [22]. And even mild depression symptoms, as classified by PHQ-9, are associated with relatively poor prognoses among cardiac patients [23]. The cut-off score of ≥10 for the detection of clinical depression has been shown to have 89% sensitivity and 89% specificity [24]. GAD-7 score ranges from 0 to 21 with a score of 0–4, 5–9, 10–14, 15–21 representing normal, mild, moderate and severe levels of anxiety, respectively. According to the widely use of the cut-off value of 5, we compared the differences between patients with GAD-7 scores of < 5 and ≥ 5. Moreover, a GAD-7 score of ≥10 indicated that the patient has clinical anxiety, in which the sensitivity and specificity of this diagnostic criterion attained 89 and 82%, respectively [25, 26]. Former studies have shown the underlying structure of GAD-7 to be unidimensional [27]. The Chinese versions of PHQ-9 and GAD-7 have been validated among Chinese cardiac patients [28, 29].

As depression and anxiety are two aspects of mental state of patients and the moderately strong inter correlation between them, we tried to use a more direct way to demonstrate the severity of comprehensive mood symptoms. We screened out patients with PHQ-9 scores of ≥5 and GAD-7 scores of ≥5 into the comorbidity subgroup, and patients with both scores ≥10 were placed in the clinical comorbidity subgroup. The combination of PHQ-9 and GAD-7 scales has been merged as a composite measure of depression and anxiety as PHQ-ADS scale [30]. And PHQ-ADS cutpoints of 10, 20, and 30 indicated mild, moderate, and severe levels of depression/anxiety, respectively. The reliability and validity of this combination and cutpoints have been examined and testified in latest studies [30–32].

### Statistical analysis

We compared the characteristics of patients with different depression and anxiety symptoms. The normality test was performed on continuous variables. In this study, the continuous values of PHQ-9 and GAD-7 scores were non-normal distributions. Clinical characteristics of patients were presented by questionnaire scores of < 5, 5–9, and ≥ 10.

The survival analysis was conducted using Kaplan–Meier survival curve and the Log-Rank test with depression and anxiety as the main determinants of the clinical outcomes. This study used cut-off 5 points to compare prognosis of patients with mood symptoms versus without mood symptoms while using cut-off 10 points to compare with clinical mood disorders versus normal participants. For statistically- or clinically-significant variables in univariate analysis, risk factors including age, gender, education level, and severity of coronary artery stenosis were included in the Cox proportional hazard regression model to adjust their effects on the prognosis. The interactions among all the covariates were considered. The HRs and 95% CIs of the endpoint events of the enrolled patients were calculated. All analyses were conducted using SPSS version 22. A *P* value of < 0.05 indicates that the difference is statistically significant.

## Results

### Prevalence and correlates of depression and anxiety

A total of 443 patients were enrolled in the study and assessed using PHQ-9 and GAD-7 scales. The baseline characteristics of the participants are shown in Table 1. The average participant was 63.9 years old (± 9.8 years), and there were 337 (76.1%) males and 106 females (23.9%). Among all the AP patients, there were 172 (38.9%) with depression symptom (PHQ-9 scores of ≥5), of which 49 (11.1%) had clinical depression (PHQ-9 scores of ≥10). As for anxiety symptoms, 103 (23.3%), 13 (2.9%) and 11 (2.5%) patients had mild, moderate and severe anxiety symptoms, respectively. Furthermore, 71 participants suffered from the comorbidity of depression and anxiety, among them, 52 (73.2%) comprised the comorbidity subgroup and 19 (26.8%) comprised clinical comorbidity subgroup. The correlation of depression and anxiety were shown in our previous article [21].

**Table 1:** Baseline characteristic and follow-up events of depression, anxiety and comorbidity in AP patients

Variates	Total *N* = 443	Depression (*N* = 172)		Anxiety (*N* = 127)		Comorbidity (*N* = 71)	
		Mild dep. (5 ≤ score < 10) *N* = 123	Mod-severe dep. (score ≥ 10) *N* = 49	Mild anx. (5 ≤ score < 10) *N* = 103	Mod-severe anx. (score ≥ 10) *N* = 24	Dep. & anx. (5 ≤ both score < 10) *N* = 52	Dep. & anx. (both scores≥10) (*N* = 19)
Age, mean ± SD, y	63.9 ± 9.8	65.5 ± 10.0	64.9 ± 10.8	63.4 ± 8.9	62.5 ± 12.7	63.85 ± 9.84	64 ± 9.8
Male, No. (%)	337(76.1)	79(64.2)	35(71.4)	63(61.2)	18(75.0)	30(57.7)	14(73.7)
Marriage, No. (%)							
Married	412(93.0)	112(91.1)	42(85.7)	96(93.2)	22(91.7)	47(90.4)	17(89.5)
Divorced or Widowed or Single	31(1.6)	11(8.9)	7(14.3)	7(6.8)	2(8.3)	5(9.6)	2(10.5)
Education, No. (%)							
Less than 6 years	115(26.0)	44(35.8)	21(42.9)	38(36.9)	10(41.7)	22(42.3)	8(42.1)
7–9 years	126(28.4)	31(25.2)	10(20.4)	28(27.2)	5(20.8)	12(23.1)	3(15.8)
10–12 years	97(21.9)	25(20.3)	9(18.4)	23(22.3)	4(16.7)	12(23.1)	3(15.8)
More than 12 years	105(23.7)	23(18.7)	9(18.4)	14(13.6)	5(20.8)	6(11.5)	5(26.3)
Severity of coronary stenosis, No.(%)							
1	93(21.0)	33(26.8)	9(18.4)	32(31.1)	5(20.8)	17(32.7)	4(21.1)
2	82(18.5)	19(15.4)	9(18.4)	11(10.7)	7(29.2)	4(7.7)	5(26.3)
3	268(60.5)	71(57.7)	31(63.3)	60(58.3)	12(50.0)	31(59.6)	10(52.6)
Events, No. (%)							
Cardiac readmission	74	21(28.4)	9(12.2)	15(20.3)	5(6.8)	9(17.3)	4(21.0)
Noncardiac readmission	46	17(37.0)	10(21.7)	8(17.4)	6(13.0)	3(5.8)	5(10.9)
Mace	88	25(20.3)	14(28.6)	18(20.5)	9(10.2)	10(19.2)	8(9.1)
Composite	129	39(31.7)	21(42.9)	24(18.6)	14(10.9)	12(23.1)	12(9.3)

*Abbreviations*: *dep.* depression, *mod-severe dep.* moderate or severe depression, *anx.* anxiety, *mod-severe anx.* moderate or severe anxiety

&: And

Total: Baseline characteristic and follow-up events of the whole cohort samples

### Follow-up events

**Fig. 1:**
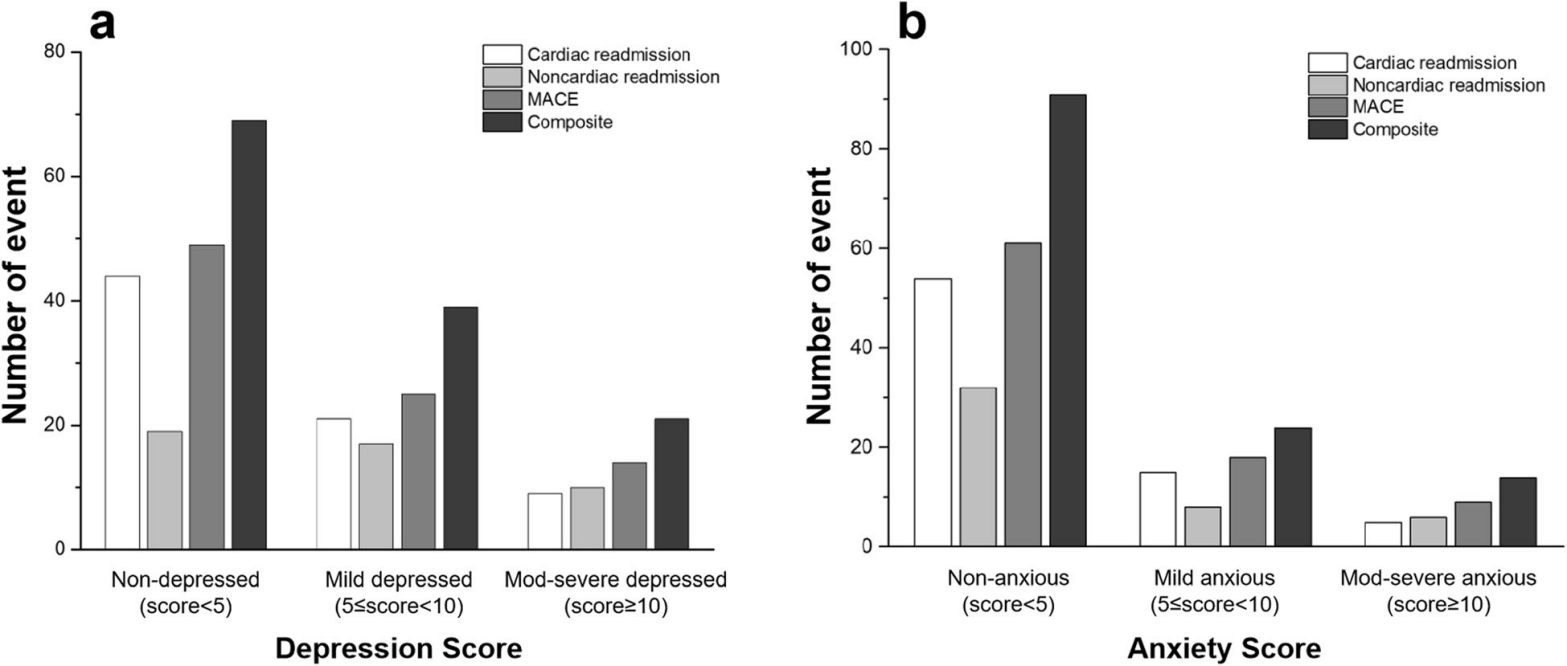
Frequency of events in different mood symptom groups. **a** is the depression group. **b** is the anxiety group

All 443 patients received follow-up calls for readmissions, MACEs, and composite events. The censored data contained loss to follow-up and endpoint events that had not occurred at the end of the study. In this study, there were 74 cardiac readmissions, 46 noncardiac readmissions, 88 MACEs and 129 composite events during the 29-month follow-up period (Fig. 1). Five patients suffered from both noncardiac readmissions and MACEs. The incidence of follow-up events in different groups is shown in Table 1.

We initially analysed prognosis of patients with PHQ-9 and GAD-7 scores of ≥5 (see Additional file 1: Table S1). Unadjusted HRs were estimated from the Kaplan–Meier survival curve and Log-Rank tests for patients without mood symptoms and with mood symptoms. Between the nondepressed and depressed group (PHQ-9 scores of ≥5), a significant difference of noncardiac readmission ([HR] 2.16, 95% [CI] 1.19–3.93, *p* = 0.011) was observed. However, depression symptoms were found to be not significantly associated with cardiac readmissions ([HR] 1.03, 95% [CI] 0.64–1.65, *p* = 0.908) and MACEs ([HR] 1.24, 95% [CI] 0.82–1.90, *p* = 0.338). Moreover, we did not observe a significant relationship between anxiety symptoms (GAD-7 scores of ≥5) and prognosis. (see Additional file 1: Table S1) As for the comorbidity subgroup, we found that coexistence of depression and anxiety may lead to more noncardiac readmission ([HR] 2.28, 95% [CI] 1.05–4.94, *p* = 0.036). A Cox proportional regression model was established for multi-factors analyses. The multi-factors included age, gender, education and severity of coronary artery stenosis, which age, gender and education were statistically significant in univariate analysis and severity of coronary artery stenosis was an important clinical indicator. Compared to patients without mood symptoms, patients with depression symptoms (PHQ-9 scores of ≥5) didn’t show a high risk of readmission ([HR] 1.81, 95% [CI] 0.55–5.96, *p* = 0.333). Meanwhile, after adjustment, we did not observe significant effects of the comorbidity (both PHQ-9 and GAD-7 scores of ≥5) on the noncardiac readmission. However, there was a tendency that patients with comorbidity may had more risks than patients with individual mood symptom on the follow-up events (see Additional file 1: Table S1).

**Fig. 2:**
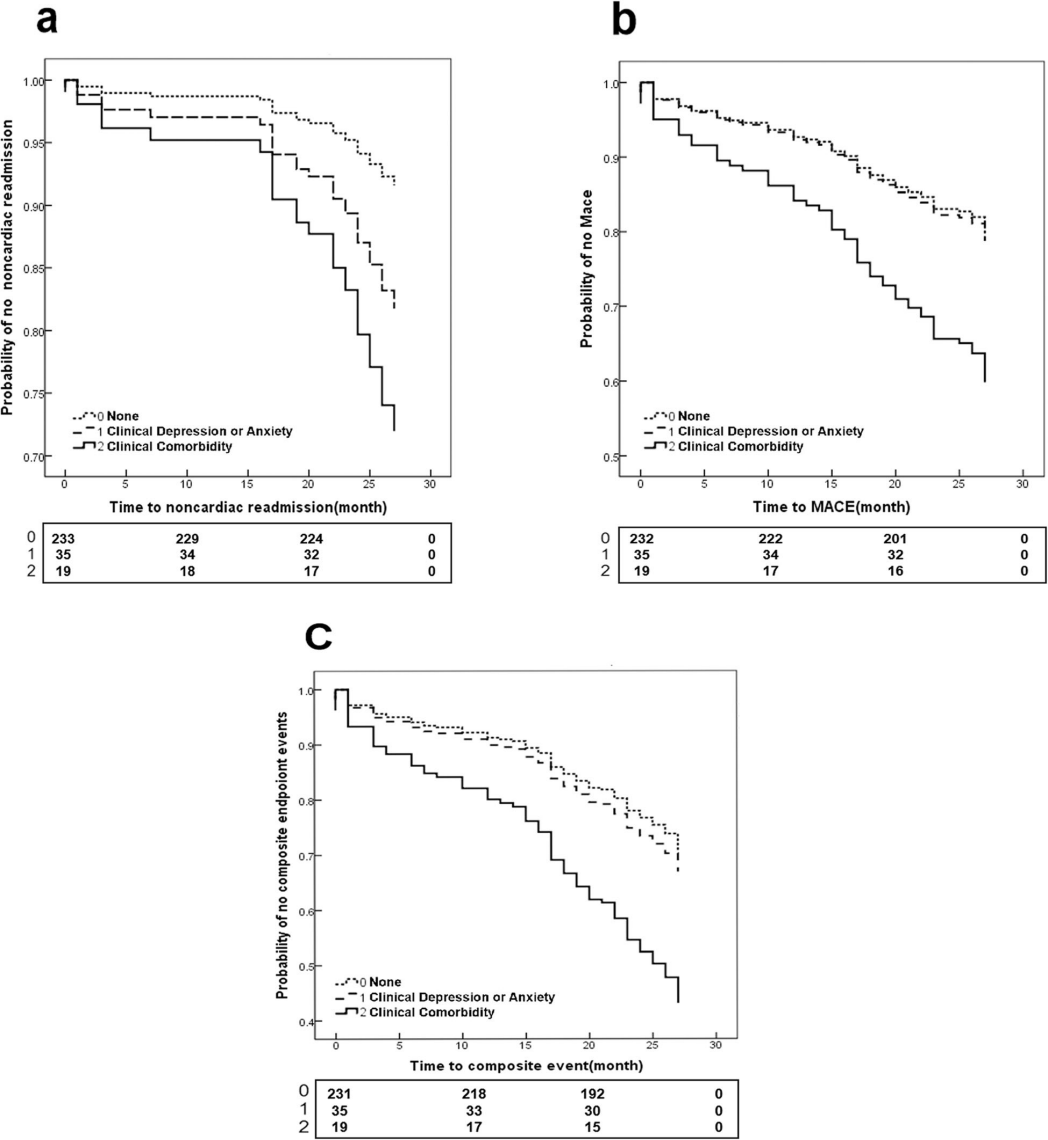
Kaplan–Meier curves of follow-up events with number of subjects at risk among the no mood symptom group, only clinical depression and anxiety group, and clinical comorbidity group. **a**, **b**, and **c** show noncardiac readmission, MACEs, and composite event, respectively. Clinical depression or anxiety: PHQ-9 or GAD-7 score of ≥10. Clinical comorbidity: Both PHQ-9 and GAD-7 scores of ≥10

Moreover, we analysed clinical mood disorders with PHQ-9 and GAD-7 scores of ≥10 (see Table 2). Compared to patients without mood disorders, patients with clinical depression (PHQ-9 scores of ≥10) had a greater risk of noncardiac readmission ([HR] 2.81, 95% [CI] 1.30–6.10, *p* = 0.009). At the same time, clinical anxiety (GAD-7 scores of ≥10) were found to have a strong significance with noncardiac readmission ([HR] 3.46, 95% [CI] 1.37–8.72, *p* = 0.008) and composite event ([HR] 2.19, 95% [CI] 1.23–3.91, p = 0.008) while MACEs were found to have a positive correlation tendency in this group ([HR] 1.99, 95% [CI] 0.97–4.07, *p* = 0.061). As for the clinical comorbidity subgroup (both PHQ-9 and GAD-7 scores of ≥10), we performed Kaplan–Meier (KM) curves of endpoint events with number of subjects at risk (Fig. 2). The unadjusted analysis showed that clinical comorbidity presented great significance with occurrence of noncardiac readmission ([HR] 3.74, 95% [CI] 1.39–10.10, *p* = 0.009), MACEs ([HR] 2.27, 95% [CI] 1.07–4.81, *p* = 0.033) and composite event ([HR] 2.44, 95% [CI] 1.31–4.52, *p* = 0.005). After controlling for age, gender, marriage and severity of coronary artery stenosis, participants with clinical depression were found to be at a greater risk of noncardiac readmission ([HR]2.38, 95% [CI] 1.06–5.33, *p* = 0.035) and composite event ([HR]1.73, 95% [CI] 1.04–2.87, *p* = 0.036) than normal samples. Meanwhile, clinical anxiety participants reported a significant risk of more MACEs ([HR] 2.23, 95% [CI] 1.07–4.64, *p* = 0.032) and composite event ([HR]2.30, 95% [CI] 1.27–4.15, *p* = 0.006) than normal participants after multi-factors adjustment. In addition, clinical comorbidity was found to be a great predictor of noncardiac readmission ([HR] 2.91, 95% [CI] 1.03–8.18, *p* = 0.043), MACEs ([HR] 2.38, 95% [CI] 1.11–5.10, *p* = 0.025) and composite events ([HR] 2. 52, 95% [CI] 1.35–4.69, *p* = 0.004) (see Table 2).

**Table 2:** Results for clinical depression, clinical anxiety and their comorbidity as different predictors of follow-up events

Event		Clinical depression				Clinical anxiety				Clinical comorbidity			
		Unadjusted HR (95%CI)	*P*	Adjusted HR (95%CI)	*P*	Unadjusted HR (95%CI)	*P*	Adjusted HR (95%CI)	*P*	Unadjusted HR (95%CI)	*P*	Adjusted HR (95%CI)	*P*
Noncardiac	Reference	2.81 (1.30–6.10)	0.009^*^	2.38 (1.06–5.33)	0.035^*^	3.46(1.37–8.72)	0.008^*^	2.85 (1.10–7.45)	0.032^*^	3.74(1.39–10.10)	0.009^*^	2.91(1.03–8.18)	0.043^*^
Cardiac	Reference	1.06 (0.52–2.19)	0.872	1.28 (0.61–2.67)	0.513	1.21(0.48–3.07)	0.684	1.39 (0.54–3.57)	0.493	1.25(0.45–3.50)	0.670	1.32(0.47–3.7)	0.604
Mace	Reference	1.53 (0.84–2.78)	0.169	1.71 (0.92–3.16)	0.089	1.99(0.97–4.07)	0.061	2.23 (1.07–4.64)	0.032^*^	2.27(1.07–4.81)	0.033^*^	2.38(1.11–5.10)	0.025^*^
Composite	Reference	1.63 (0.99–2.66)	0.054	1.73(1.04–2.87)	0.036^*^	2.19(1.23–3.91)	0.008^*^	2.30 (1.27–4.15)	0.006^*^	2.44(1.31–4.52)	0.005^*^	2.52(1.35–4.69)	0.004^*^

The table were combined with results of 3 survival analyses including clinical depression vs reference, clinical anxiety vs reference and clinical comorbidity vs reference

Reference: no depression or anxiety

Clinical Depression: PHQ-9 scores of ≥10

Clinical Anxiety: GAD-7 scores of ≥10

Clinical Comorbidity: Both PHQ-9 and GAD-7 scores of ≥10

*:*P* < 0.05

## Discussion

Following our previous cross-sectional analysis, in this study, we followed up with the patients for 1 year and over 2 years, and several clinical outcomes were observed. We found that regardless of whether multiple factors were adjusted for, patients enduring AP with depression symptoms had a significantly high rate of noncardiac readmission and incidence of composite events while AP patients with anxiety symptoms did not. People with clinical depression and anxiety were found to have great risk of noncardiac readmission and composite events. Meanwhile, clinical anxiety was found to be associated with MACEs. What we discovered but many other researchers did not pay attention to was the relationship between the clinical comorbidity of depression and anxiety and prognosis for AP patients, and we elaborated on the fact that this clinical comorbidity might be a predictor of an increased risk of noncardiac readmission, MACEs and composite events.

To our knowledge, this is the first study with an examination of the relationship between depression, anxiety, comorbidity and noncardiac readmission among AP patients. Current studies underscore the complex relationship between depression or anxiety symptoms and prognosis in patients with CVD. However, most related studies were focused on populations with myocardial infarction or heart failure, and few medical researchers pay attention to AP. Besides, the endpoint events of many previous studies were normally related to death or the reoccurrence of cardiovascular admission instead of noncardiac readmission.

### Association between depression and prognosis

In our study, patients with clinical depression symptoms (PHQ-9 score of ≥10) were found to have a 2.38 times higher risk of noncardiac readmission than those who did not suffer from depression. Pederson et al. [33] showed that depression might be an unrecognised, and possibly changeable, independent risk factor for unexpected readmission or premature death. Depression is difficult to detect in an acute care environment and is often undertreated. Patients with moderate to severe depression symptoms at discharge with no history of depression may face a twofold increase in the risk of short-term hospital readmission or death. Mitchell et al. [34] reported that the risk of hospital readmission or emergency department treatment within 30 days after discharge among patients with PHQ-9 scores of ≥5 was increased by 73%. Cancino et al. [35] showed a 49% increase in readmission within 30 days of discharge for patients with mild depression and a 96% increase for patients with moderate to severe depression. Moraska et al. [36] found that high clinical depression scores on the PHQ-9 were associated with increased risk of hospitalisation (adjusted HR: 1.79, 95% CI: 1.30–2.47). In a small study of 144 patients, Kartha et al. [37] illustrated that patients who were diagnosed with severe depression using a standardised scoring algorithm based on PHQ-9 were three times more likely than others to be rehospitalised within 90 days of discharge. We used PHQ-9 scores of < 5, 5–9 and ≥ 10 as the thresholds for non-depression, mild depression and moderate to severe depression, respectively. We also found that both mild and moderate to severe depression symptoms were associated with noncardiac readmission and composite events.

Previous literature has shown that CHD combined with depression symptoms will lead to an increase in mortality and rehospitalisation, thereby aggravating the severity of the disease and medical expenses and wasting resources. A number of studies [38] have shown that the comorbidity of mental and physical illnesses can increase the severity of disease, frequencies of readmissions and medical expenses. Even the presence of isolated mood symptoms might increase one’s risk of having medical comorbidities [39]. People with mood symptoms but no mood disorder diagnosis were found to be prone to have more lifelong diseases and health problems and longer hospital stays than those without mood diagnoses or symptoms [40]. Our study has shown that both mild depression and moderate to severe depression might be risk factors for hospital readmission.

### Association between anxiety and prognosis

Former studies have shown that anxiety is a significant risk factor for recurrent CV events and mortality [3, 13, 41–43]. Although we did not analyse death, revascularisation or nonfatal myocardial infarction independently due to a lack of valid samples, we summed up cardiac readmission, nonfatal stroke and all the above as MACEs. Tully et al. [44] found that in patients with confirmed CHD, anxiety was a prognostic risk of subsequent MACEs, such as myocardial infarction, left ventricular failure and stroke. Another study showed that generalised anxiety disorder increased the MACE risk of acute coronary syndrome outpatients by nearly two times within 2 years [45]. Similar to previous researches, we identified an association between clinical anxiety and poor CVD prognoses. Although there have been few studies in which the authors analysed the relationship between anxiety and noncardiac readmission, we found a positive correlation between clinical anxiety and noncardiac readmission. We did not observe this association in the anxiety symptom group (GAD-7 score of ≥5), possibly because the anxiety symptom is transient and usually triggered by a perceived threat, and once the threat diminishes, the anxiety will diminish too. It has been proven that anxiety is prone to reduce the quality of life of patients with CHD, especially after an acute coronary syndrome event, and that anxious patients have more disabilities and somatic symptoms than others [46–48].

### Association between comorbidity and prognosis

The authors of many related studies analysed depression or anxiety as single risk indicators, but we studied them as a comorbid symptom. We discovered that most clinical anxiety patients also have depression symptoms. Denollet and Strik et al. [41, 49] reported that a mixed emotional state consisting of anxiety and depression symptoms, not just depression, was the most common manifestation among patients with myocardial infarction [49]. The author, thus, concluded that anxiety was a common factor of depression after myocardial infarction. A similar symptom overlap was found by Frasure-Smith [45]. Some authors [50, 51] demonstrated that this connection was produced by the same dysfunctional biology or that depression and anxiety might be derived from parallel genetic dispositions. Furthermore, Scott et al. showed that comorbid depression-anxiety disorder increased the risk of a series of physical symptoms occurring at the same time [52]. In a follow-up study of women with suspected myocardial ischemia, depression or anxiety could be used to predict CVD events and death more accurately than the independent symptoms [53]. Phillip et al. [54] showed that even after adjustment, the strongest association between CVD and death from all causes appeared in major depressive disorder and generalised anxiety disorder comorbidity. Similarly, we identified a greater risk of noncardiac readmission, MACEs, and composite events among participants with both depression and anxiety.

### Limitations

First, due to the small sample size and prognostic events, some statistical analyses in this study may suffer from overfitting. Second, depression and anxiety are strongly correlated and are two aspects of mental state of patients. It would be not all-sided enough to analyse them individually. While the combined PHQ-9 ≥ 5 & GAD-7 ≥ 5 and PHQ-9 ≥ 10 & GAD-7 ≥ 10 has not been strictly validated, there were some researches have already tried to testify this combination [55, 56]. Although we did not carry out professional psychiatric interviews to confirm the diagnosis of mood symptoms, we used an easy-to-deploy screening tool that has been fully validated. Moreover, our research was conducted in only one hospital specialised in cardiac care, which ensured the consistency of examination tools, methods and follow-up times and procedures but limited the generalizability of the findings.

## Conclusion

AP patients with clinical depression disorders tend to have a higher incidence of noncardiac readmission, and composite events than others. Clinical anxiety patients were found to have an increased risk of noncardiac readmission, and MACEs. Besides, clinical comorbidity of depression and anxiety may lead to a worse prognosis than one disorder alone. How clinicians understand this overlap of symptoms will help them determine diagnoses, treatment options, and possible referrals for additional testing and services. In the future, researchers should use effective and reliable measurement methods to further examine the relationship between depression, anxiety, and CHD while paying close attention to the overlapping symptoms.

## Supplementary Information

**Additional file 1: Table S1**. Results for depression symptom, anxiety symptom and their comorbidity as different predictors of follow-up events.

**Additional file 2: Table S2**. Results for depression, anxiety by 75% quartile of PHQ-9 and GAD-7 score as predictors of follow-up events.

## Data Availability

The datasets obtained and/or analysed during the current study are available from the corresponding author on reasonable request.

## Abbreviations

CVD: Cardiovascular disease
AP: Angina pectoris
MACE: Major cardiovascular event
PHQ: Patient health questionnaire
GAD: Generalised anxiety disorder
HR: Hazard ratio
CI: Confidence interval
CHD: Coronary heart disease
CV: Cardiovascular
KM: Kaplan–Meier

## References

[1] Lett HS, Blumenthal JA, Babyak MA, Sherwood A, Strauman T, Robins C, Newman MF (2004). Depression as a risk factor for coronary artery disease: evidence, mechanisms, and treatment. Psychosom Med.

[2] Denollet J, Schiffer AA, Spek V (2010). A general propensity to psychological distress affects cardiovascular outcomes: evidence from research on the type D (distressed) personality profile. Circ Cardiovasc Qual Outcomes.

[3] Roest AM, Martens EJ, de Jonge P, Denollet J (2010). Anxiety and risk of incident coronary heart disease: a meta-analysis. J Am Coll Cardiol.

[4] Leung YW, Flora DB, Gravely S, Irvine J, Carney RM, Grace SL (2012). The impact of premorbid and postmorbid depression onset on mortality and cardiac morbidity among patients with coronary heart disease: meta-analysis. Psychosom Med.

[5] Meijer A, Conradi HJ, Bos EH, Anselmino M, Carney RM, Denollet J, Doyle F, Freedland KE, Grace SL, Hosseini SH, Lane DA, Pilote L, Parakh K, Rafanelli C, Sato H, Steeds RP, Welin C, de Jonge P (2013). Adjusted prognostic association of depression following myocardial infarction with mortality and cardiovascular events: individual patient data meta-analysis. Br J Psychiatry.

[6] Lichtman JH, Froelicher ES, Blumenthal JA, Carney RM, Doering LV, Frasure-Smith N, Freedland KE, Jaffe AS, Leifheit-Limson EC, Sheps DS, Vaccarino V, Wulsin L, American Heart Association Statistics Committee of the Council on Epidemiology and Prevention and the Council on Cardiovascular and Stroke Nursing (2014). Depression as a risk factor for poor prognosis among patients with acute coronary syndrome: systematic review and recommendations: a scientific statement from the American Heart Association. Circulation..

[7] Jha MK, Qamar A, Vaduganathan M, Charney DS, Murrough JW (2019). Screening and management of depression in patients with cardiovascular disease: JACC state-of-the-art review. J Am Coll Cardiol.

[8] Nicholson A, Kuper H, Hemingway H (2006). Depression as an aetiologic and prognostic factor in coronary heart disease: a meta-analysis of 6362 events among 146 538 participants in 54 observational studies. Eur Heart J.

[9] Barth J, Schumacher M, Herrmann-Lingen C (2004). Depression as a risk factor for mortality in patients with coronary heart disease: a meta-analysis. Psychosom Med.

[10] van Melle JP, de Jonge P, Spijkerman TA, Tijssen JGP, Ormel J, van Veldhuisen DJ, van den Brink RHS, van den Berg MP (2004). Prognostic association of depression following myocardial infarction with mortality and cardiovascular events: a meta-analysis. Psychosom Med.

[11] Grewal K, Gravely-Witte S, Stewart DE, Grace SL (2011). A simultaneous test of the relationship between identified psychosocial risk factors and recurrent events in coronary artery disease patients. Anxiety Stress Coping.

[12] Weissman MM, Markowitz JS, Ouellette R, Greenwald S, Kahn JP (1990). Panic disorder and cardiovascular/cerebrovascular problems: results from a community survey. Am J Psychiatry.

[13] Grace SL, Abbey SE, Irvine J, Shnek ZM, Stewart DE (2004). Prospective examination of anxiety persistence and its relationship to cardiac symptoms and recurrent cardiac events. Psychother Psychosom.

[14] Eaker ED, Sullivan LM, Kelly-Hayes M, D’Agostino RB, Benjamin EJ (2005). Tension and anxiety and the prediction of the 10-year incidence of coronary heart disease, atrial fibrillation, and total mortality: the Framingham Offspring Study. Psychosom Med.

[15] Kurdyak PA, Gnam WH, Goering P, Chong A, Alter DA (2008). The relationship between depressive symptoms, health service consumption, and prognosis after acute myocardial infarction: a prospective cohort study. BMC Health Serv Res.

[16] May HT, Horne BD, Carlquist JF, Sheng X, Joy E, Catinella AP (2009). Depression after coronary artery disease is associated with heart failure. J Am Coll Cardiol.

[17] Kessler RC, DuPont RL, Berglund P, Wittchen HU (1999). Impairment in pure and comorbid generalized anxiety disorder and major depression at 12 months in two national surveys. Am J Psychiatry.

[18] Kessler RC, Chiu WT, Demler O, Merikangas KR, Walters EE (2005). Prevalence, severity, and comorbidity of 12-month DSM-IV disorders in the National Comorbidity Survey Replication. Arch Gen Psychiatry.

[19] Suls J, Bunde J (2005). Anger, anxiety, and depression as risk factors for cardiovascular disease: the problems and implications of overlapping affective dispositions. Psychol Bull.

[20] Sevincok L, Buyukozturk A, Dereboy F (2001). Serum lipid concentrations in patients with comorbid generalized anxiety disorder and major depressive disorder. Can J Psychiatr.

[21] Yin H, Liu Y, Ma H, Liu G, Guo L, Geng Q (2019). Associations of mood symptoms with NYHA functional classes in angina pectoris patients: a cross-sectional study. BMC Psychiatry.

[22] Kroenke K, Spitzer RL, Williams JB (2001). The PHQ-9: validity of a brief depression severity measure. J Gen Intern Med.

[23] Manea L, Gilbody S, McMillan D (2012). Optimal cut-off score for diagnosing depression with the patient health questionnaire (PHQ-9): a meta-analysis. CMAJ..

[24] Leavens A, Patten SB, Hudson M, Baron M, Thombs BD, Canadian Scleroderma Research Group (2012). Influence of somatic symptoms on Patient Health Questionnaire-9 depression scores among patients with systemic sclerosis compared to a healthy general population sample. Arthritis Care Res.

[25] Spitzer RL, Kroenke K, Williams JB, Löwe B (2006). A brief measure for assessing generalized anxiety disorder: the GAD-7. Arch Intern Med.

[26] Swinson RP (2006). The GAD-7 scale was accurate for diagnosing generalised anxiety disorder. Evid Based Med.

[27] Sawaya H, Atoui M, Hamadeh A, Zeinoun P, Nahas Z (2016). Adaptation and initial validation of the patient health questionnaire - 9 (PHQ-9) and the generalized anxiety disorder - 7 questionnaire (GAD-7) in an Arabic speaking Lebanese psychiatric outpatient sample. Psychiatry Res.

[28] Zhu Y, Blumenthal JA, Shi C, Jiang R, Patel A, Zhang A, Yu X, Gao R, Wu Y (2018). Sedentary behavior and the risk of depression in patients with acute coronary syndromes. Am J Cardiol.

[29] Wang L, Lu K, Wang C, Sheng L, Hu D, Rongjing D (2014). Reliability and validity of GAD-2 and GAD-7 for anxiety screening in cardiovascular disease clinic. Sichuan Ment Health.

[30] Kroenke K, Wu J, Yu Z, Bair MJ, Kean J, Stump T, Monahan PO (2016). Patient health questionnaire anxiety and depression scale: initial validation in three clinical trials. Psychosom Med.

[31] Chilcot J, Hudson JL, Moss-Morris R, Carroll A, Game D, Simpson A, Hotopf M (2018). Screening for psychological distress using the patient health questionnaire anxiety and depression scale (PHQ-ADS): initial validation of structural validity in dialysis patients. Gen Hosp Psychiatry.

[32] Kroenke K, Spitzer RL, Williams JB, Löwe B (2010). The patient health questionnaire somatic, anxiety, and depressive symptom scales: a systematic review. Gen Hosp Psychiatry.

[33] Pederson JL, Majumdar SR, Forhan M, Johnson JA, McAlister FA, PROACTIVE Investigators (2016). Current depressive symptoms but not history of depression predict hospital readmission or death after discharge from medical wards: a multisite prospective cohort study. Gen Hosp Psychiatry.

[34] Mitchell SE, Paasche-Orlow MK, Forsythe SR, Chetty VK, O'Donnell JK, Greenwald JL, Culpepper L, Jack BW (2010). Post-discharge hospital utilization among adult medical inpatients with depressive symptoms. J Hosp Med.

[35] Cancino RS, Culpepper L, Sadikova E, Martin J, Jack BW, Mitchell SE (2014). Dose-response relationship between depressive symptoms and hospital readmission. J Hosp Med.

[36] Moraska AR, Chamberlain AM, Shah ND, Vickers KS, Rummans TA, Dunlay SM, Spertus JA, Weston SA, McNallan SM, Redfield MM, Roger VL (2013). Depression, healthcare utilization, and death in heart failure: a community study. Circ Heart Fail.

[37] Kartha A, Anthony D, Manasseh CS, Greenwald JL, Chetty VK, Bugess JF, Culpepper L, Jack BW (2007). Depression is a risk factor for rehospitalization in medical inpatients. Prim Care Companion J Clin Psychiatry.

[38] Baumeister H, Haschke A, Munzinger M, Hutter N, Tully PJ (2015). Inpatient and outpatient costs in patients with coronary artery disease and mental disorders: a systematic review. Biopsychosoc Med.

[39] Moreno C, Nuevo R, Chatterji S, Verdes E, Arango C, Ayuso-Mateos JL (2013). Psychotic symptoms are associated with physical health problems independently of a mental disorder diagnosis: results from the WHO world health survey. World Psychiatry.

[40] Moussavi S, Chatterji S, Verdes E, Tandon A, Patel V, Ustun B (2007). Depression, chronic diseases, and decrements in health: results from the world health surveys. Lancet..

[41] Strik JJ, Denollet J, Lousberg R, Honig A (2003). Comparing symptoms of depression and anxiety as predictors of cardiac events and increased health care consumption after myocardial infarction. J Am Coll Cardiol.

[42] Shibeshi WA, Young-Xu Y, Blatt CM (2007). Anxiety worsens prognosis in patients with coronary artery disease. J Am Coll Cardiol.

[43] Moser DK, McKinley S, Riegel B, Doering LV, Meischke H, Pelter M, Davidson P, Baker H, Dracup K (2011). Relationship of persistent symptoms of anxiety to morbidity and mortality outcomes in patients with coronary heart disease. Psychosom Med.

[44] Tully PJ, Cosh SM, Baumeister H (2014). The anxious heart in whose mind? A systematic review and meta-regression of factors associated with anxiety disorder diagnosis, treatment and morbidity risk in coronary heart disease. J Psychosom Res.

[45] Frasure-Smith N, Lespérance F (2008). Depression and anxiety as predictors of 2-year cardiac events in patients with stable coronary artery disease. Arch Gen Psychiatry.

[46] Lane D, Carroll D, Ring C, Beevers DG, Lip GY (2001). Predictors of attendance at cardiac rehabilitation after myocardial infarction. J Psychosom Res.

[47] Lane D, Carroll D, Ring C, Beevers DG, Lip GY (2001). Mortality and quality of life 12 months after myocardial infarction: effects of depression and anxiety. Psychosom Med.

[48] Müller-Tasch T, Frankenstein L, Holzapfel N, Schellberg D, Löwe B, Nelles M, Zugck C, Katus H, Rauch B, Haass M, Jünger J, Remppis A, Herzog W (2008). Panic disorder in patients with chronic heart failure. J Psychosom Res.

[49] Denollet J, Strik JJ, Lousberg R, Honig A (2006). Recognizing increased risk of depressive comorbidity after myocardial infarction: looking for 4 symptoms of anxiety-depression. Psychother Psychosom.

[50] Agorastos A, Lederbogen F, Otte C. [Treatment of depression in coronary heart disease]. Nervenarzt. 2015. 86(3): 375–385; quiz 386-7, DOI: 10.1007/s00115-014-4162-z. 10.1007/s00115-014-4162-z 25737494

[51] Abdelbasset WK, Alqahtani BA (2019). A randomized controlled trial on the impact of moderate-intensity continuous aerobic exercise on the depression status of middle-aged patients with congestive heart failure. Medicine (Baltimore).

[52] Scott KM, Bruffaerts R, Tsang A, Ormel J, Alonso J, Angermeyer MC, Benjet C, Bromet E, de Girolamo G, de Graaf R, Gasquet I, Gureje O, Haro JM, He Y, Kessler RC, Levinson D, Mneimneh ZN, Oakley Browne MA, Posada-Villa J, Stein DJ, Takeshima T, von Korff M (2007). Depression-anxiety relationships with chronic physical conditions: results from the world mental health surveys. J Affect Disord.

[53] Rutledge T, Linke SE, Krantz DS, Johnson BD, Bittner V, Eastwood JA, Eteiba W, Pepine CJ, Vaccarino V, Francis J, Vido DA, Bairey Merz CN (2009). Comorbid depression and anxiety symptoms as predictors of cardiovascular events: results from the NHLBI-sponsored Women’s Ischemia Syndrome Evaluation (WISE) study. Psychosom Med.

[54] Phillips AC, Batty GD, Gale CR, Deary IJ, Osborn D, MacIntyre K, Carroll D (2009). Generalized anxiety disorder, major depressive disorder, and their comorbidity as predictors of all-cause and cardiovascular mortality: the Vietnam experience study. Psychosom Med.

[55] Nefs G, Hendrieckx C, Reddy P, Browne JL, Bot M, Dixon J, Kyrios M, Speight J, Pouwer F (2019). Comorbid elevated symptoms of anxiety and depression in adults with type 1 or type 2 diabetes: results from the International Diabetes MILES Study. J Diabetes Complicat.

[56] Mishkin K, Nugmanova Z, Urbaeva J, Nugumanova G, Abdumananova M, Kim E, Lazariu V, McNutt LA (2021). Anxiety and depression among women living with HIV in Kazakhstan. AIDS Care.

