# Supplementary material for: Comorbidity of depression and anxiety leads to a poor prognosis following angina pectoris patients: a prospective study
Source: BMC Psychiatry. 2021 Apr 20;21:202. doi: 10.1186/s12888-021-03202-5 (PMC8056494; doi:10.1186/s12888-021-03202-5)
Supplement: Supplementary file 1 — Additional file 1: Table S1. Results for depression symptom, anxiety symptom and their comorbidity as different predictors of follow-up events. [file 12888_2021_3202_MOESM1_ESM.docx]

Table S1: Results for depression symptom, anxiety symptom and their comorbidity as different predictors of follow-up events.

| Event | Reference | Depression Symptom | | | |  | Anxiety Symptom | | | |  | Comorbidity | | | |
| --- | --- | --- | --- | --- | --- | --- | --- | --- | --- | --- | --- | --- | --- | --- | --- |
|  |  | Unadjusted HR  (95%CI) | P | Adjusted HR  (95%CI) | P |  | Unadjusted HR  (95%CI) | P | Adjusted HR  (95%CI) | P |  | Unadjusted HR  (95%CI) | P | Adjusted HR  (95%CI) | P |
| Noncardiac | Reference | 2.16(1.19-3.93) | 0.011^*^ | 1.81(0.55-5.96) | 0.333 |  | 1.45(0.72-2.92) | 0.296 | 1.22(0.57-2.58) | 0.610 |  | 2.28(1.05-4.94) | 0.036^*^ | 1.85(0.81-4.21) | 0.143 |
| Cardiac | Reference | 1.03(0.64-1.65) | 0.908 | 2.84 (0.68-11.94) | 0.153 |  | 0.92(0.54-1.58) | 0.766 | 1.10(0.63-1.92) | 0.740 |  | 1.05(0.59-1.89) | 0.861 | 1.20 (0.66-2.20) | 0.550 |
| Mace | Reference | 1.24(0.82-1.90) | 0.338 | 1.20(0.70-5.54) | 0.198 |  | 1.14(0.71-1.84) | 0.597 | 1.30(0.79-2.14) | 0.301 |  | 1.32(0.79-2.21) | 0.287 | 1.52 (0.89-2.60) | 0.123 |
| Composite | Reference | 1.35(0.95-1.92) | 0.100 | 1.77(0.81-3.88) | 0.156 |  | 1.10(0.73-1.64) | 0.651 | 1.17(0.76-1.78) | 0.476 |  | 1.34(0.88-2.06) | 0.176 | 1.46(0.93-2.29) | 0.096 |

The table were combined with results of 3 survival analyses including depression symptom vs reference, anxiety symptom vs reference and comorbidity vs reference.

*:P<0.05

Reference: No depression or anxiety.

Depression Symptom: PHQ-9 scores of ≥ 5.

Anxiety Symptom: GAD-7 scores of ≥ 5.

Comorbidity: Both PHQ-9 and GAD-7 scores of ≥ 5.
