# Supplementary material for: Comorbidity of depression and anxiety leads to a poor prognosis following angina pectoris patients: a prospective study
Source: BMC Psychiatry. 2021 Apr 20;21:202. doi: 10.1186/s12888-021-03202-5 (PMC8056494; doi:10.1186/s12888-021-03202-5)
Supplement: Supplementary file 2 — Additional file 2: Table S2. Results for depression, anxiety by 75% quartile of PHQ-9 and GAD-7 score as predictors of follow-up events. [file 12888_2021_3202_MOESM2_ESM.docx]

Table S2: Results for depression, anxiety by 75% quartile of PHQ-9 and GAD-7 score as predictors of follow-up events.

| Event | Depression | | | | | |  | Anxiety | | | | | |
| --- | --- | --- | --- | --- | --- | --- | --- | --- | --- | --- | --- | --- | --- |
|  | Unadjusted HR | 95%CI | P | Adjusted HR | 95%CI | P |  | Unadjusted HR | 95%CI | P | Adjusted HR | 95%CI | P |
| Noncardiac | 2.32 | 1.04-5.21 | 0.041^*^ | 2.00 | 0.88-4.55 | 0.098 |  | 1.22 | 0.87-3.55 | 0.628 | 1.11 | 0.49-2.55 | 0.799 |
| Cardiac | 0.93 | 0.47-1.83 | 0.832 | 1.07 | 0.54-2.14 | 0.838 |  | 0.79 | 0.41-1.51 | 0.468 | 0.87 | 0.44-1.69 | 0.674 |
| Mace | 1.21 | 0.66-2.21 | 0.545 | 1.28 | 0.69-2.37 | 0.431 |  | 0.96 | 0.54-1.70 | 0.894 | 1.06 | 0.59-1.91 | 0.847 |
| Composite | 1.38 | 0.85-2.24 | 0.194 | 1.02 | 0.86-1.20 | 0.216 |  | 0.95 | 0.60-1.52 | 0.830 | 0.98 | 0.60-1.59 | 0.935 |

*:P<0.05

25%, 50%, 75% of PHQ-9 scores are 1, 3, 6.

25%, 50%, 75% of GAD-7 scores are 1, 2, 5.
